# Case report: Acute audiovestibular presentation following hemi-pontine infarction

**DOI:** 10.3389/fstro.2023.1272796

**Published:** 2023-12-11

**Authors:** Nehzat Koohi, Salman Haider, Natallia Kharytaniuk, David J Werring, Doris-Eva Bamiou, Diego Kaski

**Affiliations:** ^1^Department of Clinical and Movement Neurosciences, University College London, Queen Square Institute of Neurology, London, United Kingdom; ^2^Comprehensive Stroke Service, National Hospital for Neurology and Neurosurgery, London, United Kingdom; ^3^The Ear Institute, University College London, London, United Kingdom; ^4^Department of Neurootology, Royal ENT and Eastman Dental Hospitals, London, United Kingdom; ^5^Department of Brain Repair and Rehabilitation, Stroke Research Centre, University College London Queen Square Institute of Neurology, London, United Kingdom; ^6^National Institute for Health and Care Research, University College London Hospitals Biomedical Research Centre (Deafness and Hearing Problems Theme), London, United Kingdom

**Keywords:** AICA, pons, infarction, acute vertigo, unilateral hearing loss

## Abstract

Posterior circulation stroke is an uncommon cause of sudden-onset sensorineural hearing loss (SSNHL). Anterior inferior cerebellar artery occlusion results in ipsilateral peripheral audiovestibular dysfunction. Few reports describe posterior circulation stroke presenting with contralateral SSNHL and acute vestibular and focal neurological signs. We present a case of contralateral SSNHL and isolated acute central vestibular dysfunction in the absence of other central focal neurological deficits. To the best of our knowledge, this has not been described to date. The patient was identified to have asymmetrical SSNHL, subtle skew deviation with left head tilt, and significant refixation saccades on video head impulse test despite bilaterally normal vestibulo-ocular reflex gains. Left pontine infarct was suspected and confirmed on magnetic resonance imaging. The patient was treated with an appropriate antiplatelet regimen. We highlight the importance of a thorough clinical diagnostic work-up as posterior circulation strokes with isolated audiovestibular deficits can be easily missed if other significant neurological deficits are absent.

## Introduction

Sudden-onset sensorineural hearing loss (SSNHL) is commonly unilateral and often accompanied by tinnitus and, in some cases, vertigo (Chau et al., 2010). Although a myriad of causes has been proposed, including infective, vascular, immune, and others (Schreiber et al., 2010), acute SSNHL caused by an ischaemic stroke is uncommon. Vascular SSNHL is usually the result of an infarction in the posterior circulation territory, namely, occlusion of the anterior inferior cerebellar artery (AICA) (Kim and Lee, 2017). The blood supply to the audiovestibular peripheral apparatus arises from the AICA, with the ischaemic event producing a “peripheral” pattern of audiovestibular loss ipsilateral to the AICA territory infarct (Lee et al., 2002). There are but a few case reports of contralateral hearing loss and acute vestibular signs resulting from pontine infarction (Doyle et al., 1996; Lee and Baloh, 2005; Murakami et al., 2005). All previously reported cases describe apparent acute focal neurological deficits [e.g., diplopia, facial palsy, cerebellar ataxia, limb dysmetria, etc. (Doyle et al., 1996; Lee and Baloh, 2005; Murakami et al., 2005)], and none, to our knowledge, reported unilateral hearing loss with acute vestibular signs without other central focal neurological signs. Herein, we report a case of pontine infarction in a patient presenting with a unilateral SSNHL (contralateral to infarction) and subtle abnormal vestibular signs mimicking labyrinthitis (a rare condition).

## Case description

### Clinical history

#### Initial (baseline) presentation

A 57-year-old retired man of Asian heritage presented to the emergency department (ED) with vertigo, unsteadiness, intrusive tinnitus, and hearing loss in his right ear of sudden onset—after waking up in the morning. He had nausea but no vomiting. On direct questioning, the patient reported feeling unsteady and that he fell when attempting to stand up. He also reported heaviness and tingling in his right shoulder and upper arm. His medical history included type 2 diabetes mellitus, hypertension, chronic back pain, depression, and anxiety. He practices yoga regularly to manage his anxiety and depression. He had never smoked or consumed alcohol.

On arrival to our acute neurovascular unit, embedded within the ED at University College London Hospital 5 h after his symptom onset, his blood pressure was 171/95 mm Hg. A 12-lead electrocardiogram (ECG) showed signs of left atrial enlargement (total *P* wave duration >60 ms); however, a subsequent cardiac work-up (transthoracic echo) ruled out any significant cardioembolic cause for the acute cerebrovascular event. The initial ED neurological examination, including tone, muscle strength, reflexes, coordination, and sensation, was reported normal. Laboratory investigations including hematological analysis and renal, liver, and lipid profiles were unremarkable, except for the patient's HbA1c level, which was mildly elevated, suggestive of suboptimal glycaemic control. A computed tomography (CT) scan of the head (including a CT angiogram), performed within 24 h of the symptom onset, was unremarkable. Subsequent neurological examination by the stroke team confirmed right shoulder and upper arm paraesthesia without associated sensory deficits. The remainder of the neurological examination was normal. The stroke team's assessment did not identify any ocular motor deficits. An initial diagnosis of labyrinthitis was speculated on the basis of the hearing loss and vertigo, and the patient was referred to our acute vertigo specialist team as part of the acute neurovascular service for further evaluation. Stroke was considered possible due to the presence of subtle additional neurological signs (gait ataxia and sensory symptoms).

## Diagnostic assessment

### Audiovestibular investigations

**Figure 1:**
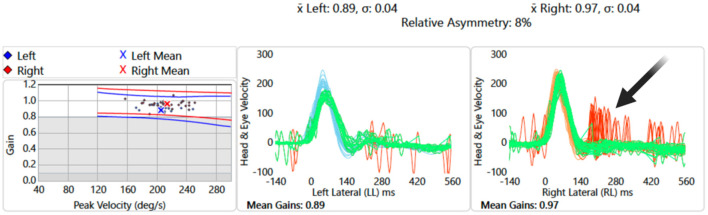
Video head impulse test of lateral semicircular canals: data showing overt “refixation” saccades on the right (red traces, black arrow) despite normal vestibular-ocular reflex gain values.

A neuro-otological examination, performed on the same day, revealed an unsteady gait [grade I ataxia (Lee et al., 2006)]. An automated HINTS Plus (head impulse, nystagmus, test of skew, plus hearing assessement), recorded with video oculography, was performed. The results showed a subtle left-beating horizontal nystagmus. The video head impulse test (vHIT) demonstrated vestibular-ocular reflex (VOR) gain within the normal range bilaterally; however, significant “refixation” saccades were observed on the right vHIT (Figure 1), suggesting a central vestibulopathy (Koohi et al., 2022; Curthoys et al., 2023). A subtle skew deviation and a head tilt to the left were also noted. There was no occluding wax in the ear canals. Hearing screening at frequencies 1,000 and 3,000 Hz was passed on the left but failed on the right at 40 dB HL. Overall, the HINTS Plus examination results were indicative of a central pathology.

**Figure 2:**
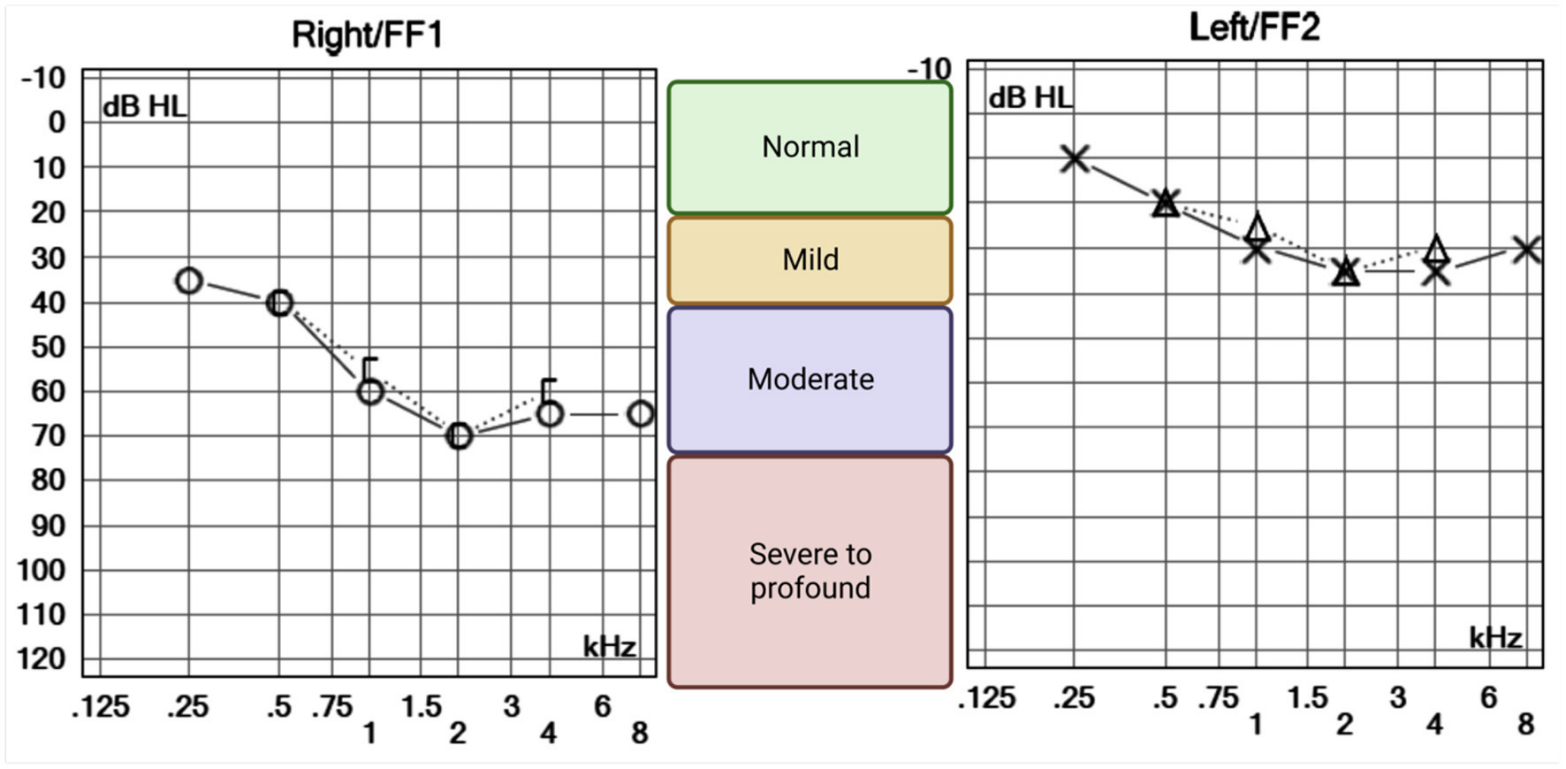
Pure tone audiometry of the right and left ears. O and X symbols represent the lowest tone perceived (decibels hearing level, dB HL) by the patient for each frequency, in kilohertz (kHz). Air (right = O and left = X) and bone (right = [ and left = Δ) conducted sounds were equally perceived suggesting a sensorineural pattern of hearing loss. High frequencies were bilaterally affected, suggesting an age-related process. However, the right ear is clearly more impaired, with lower hearing thresholds across all frequencies.

Dix–Hallpike and roll test maneuvers induced no nystagmus or relevant symptoms. Pure tone audiometry, performed 48 h after the symptom onset, revealed mild high-frequency sensorineural hearing loss in the left ear likely to be age-related and mild to moderate high-frequency sensorineural hearing loss in the right ear of pronounced asymmetry compared to the left (Figure 2).

We did not perform the caloric test as it is not standard practice in acute settings. As shown by Morrison et al. (2022), the caloric test is less precise than the vHIT for distinguishing between strokes and peripheral vestibulopathy in cases of sudden dizziness.

### Imaging and further work-up

The presence of subtle skew deviation and significant “refixation” saccades seen on the vHIT (central HINTS Plus), together with sensory appendicular symptoms were not compatible with a peripheral cause therefore further imaging was recommended to rule out an acute central pathology.

**Figure 3:**
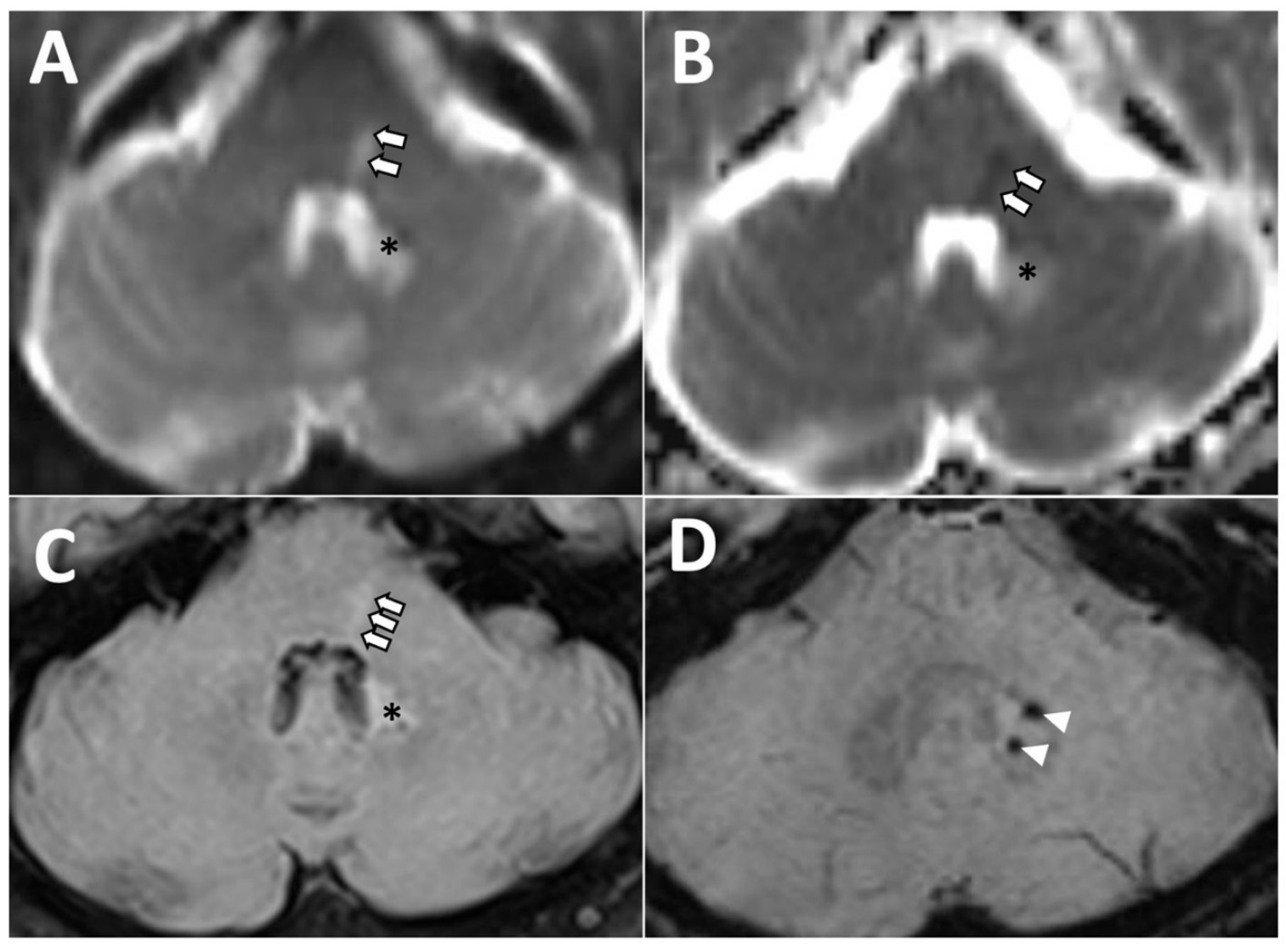
Axial magnetic resonance images of the posterior fossa demonstrating left dorsal hemi-pontine signal change (arrows) on diffusion-weighted **(A)**, apparent diffusion coefficient **(B)**, and fluid-attenuated inversion recovery (FLAIR) **(C)**, consistent with acute ischaemia in the territory of a perforating artery, and likely involving left lateral lemniscus and abducens nucleus. *Hyperintensity area associated with two microbleeds, as visualized on FLAIR **(C)** and susceptibility-weighted [arrowheads, **(D)**] images reflecting previous hemorrhage and gliotic changes.

A brain MRI (magnetic resonance imaging) scan, including diffusion-weighted imaging, performed 48 h after the symptom onset revealed a small acute infarct within the dorsal left hemi-pons (Figure 3). A small, acute right thalamic infarct was also identified. The number and distribution of white matter hyperintensities on fluid-attenuated inversion recovery imaging suggested a moderate burden of small vessel disease, with multiple scattered foci of micro hemorrhages within the temporal lobes, periventricular white matter, right caudate head, and left dentate nucleus on susceptibility-weighted images.

A 7-day ECG trace showed no atrial fibrillation, while ambulatory blood pressure monitoring revealed slightly elevated blood pressure.

### Therapeutic interventions and outcomes

Treatment for the stroke was initiated according to current guidelines on the use of antiplatelet therapy for secondary stroke prevention. Thus, dual-antiplatelet therapy was administered for 21 days, followed by the long-term use of clopidogrel (Kleindorfer et al., 2021) and daily amlodipine for blood pressure control. Three months later, his main symptoms were general weakness and fatigue; however, his balance and hearing improved such that he was mobile without assistance and able to perform regular light exercise such as yoga.

## Follow-up

An audioverstibular assessment at follow-up (17 months) showed normal distortion product otoacoustic emissions bilaterally (present at all frequencies); the auditory brainstem responses were present and normal on the right and were abnormal (absent wave V with present waves I and III) on the left. Repeat pure-tone audiometry showed improved hearing thresholds in the right ear, while a vHIT demonstrated improved VOR. Subsequent imaging (Brain MRI) was performed 18 months later and showed findings consistent with hypertensive arteriopathy.

**Figure 4:**
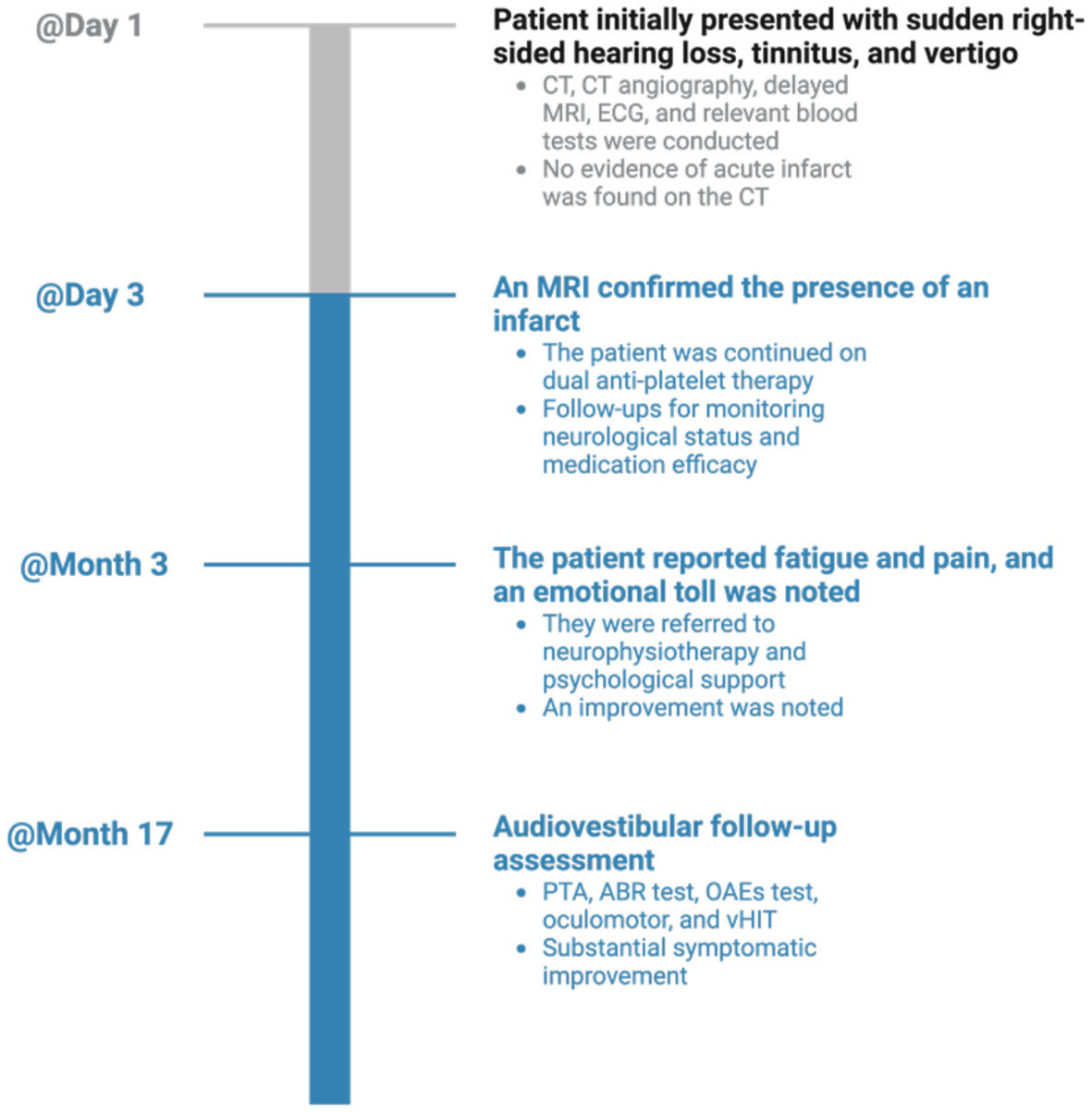
Timeline visualization of important dates/times and relevant data in the case. CT, computed tomography; MRI, magnetic resonance imaging; ECG, electrocardiogram; PTA, pure-tone audiometry; OAEs, otoacoustic emissions; ABR, auditory-evoked brainstem responses; vHIT, video head impulse test.

The case timeline is presented in Figure 4.

## Discussion

**Figure 5:**
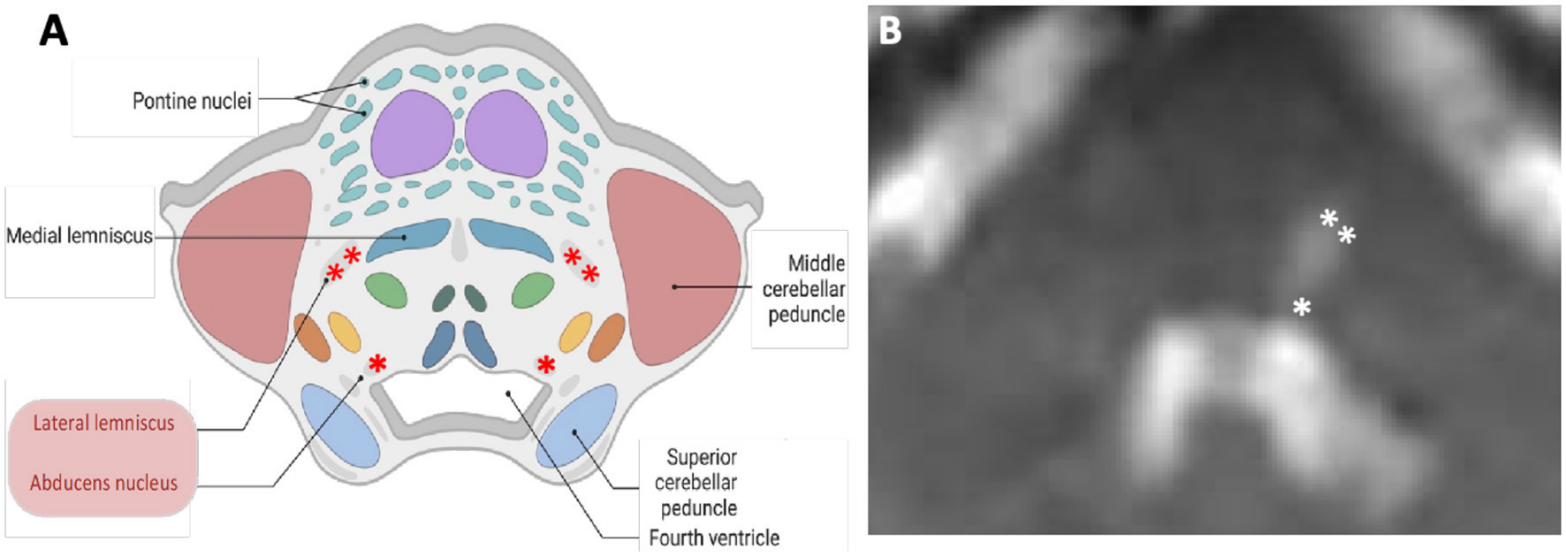
Schematic **(A)** and corresponding diffusion-weighted magnetic resonance **(B)** images of the axial cross section at the level of pons. Contralateral hearing loss from pontine ischaemia is most likely due to the damage to the left lateral lemniscus (double asterisks). The “refixation” saccades and the left gaze-evoked nystagmus are possibly due to the damage to the left abducens nucleus (single asterisk). In this case, an impaired left abducens nerve (abducens nerve palsy) resulted in an inability to transmit signals to the left lateral rectus.

We describe a patient with SSNHL and acute vertigo in whom the presence of subtle central neurological features (predominately ocular motor) facilitated the diagnosis of a pontine infarct contralateral to the SSNHL. Strokes that present with isolated audiovestibular symptoms involving posterior circulation can easily be missed (Banerjee et al., 2018) in the absence of the typical motor or language deficits that usually raise the suspicion of stroke. Early diagnosis of brainstem infarction is important to help mitigate its associated high morbidity and mortality (Banerjee et al., 2018). Detailed neuro-otological assessments can help identify central features that render a peripheral (inner-ear) etiology of hearing loss and vertigo less likely than stroke as seen in our case. SSNHL that is unilateral and isolated or when accompanied by vertigo is often attributed to labyrinthitis (a rare condition) (Barkwill and Arora, 2023). Hearing loss resulting from neural lesions, including the auditory nerve and central auditory pathway lesions (cochlear nuclei to the temporal cortex), is less frequent (Kumar et al., 1986). The anatomical limits of the infarcted region within the pons dictate the clinical syndrome, and among its various clinical presentations, cerebrovascular infarction of the central auditory pathways has been described infrequently (Muttikkal et al., 2014). In the ascending auditory pathways, fibers from the contralateral auditory nucleus in the medulla join the lateral lemniscus in the pons (Figure 5), pass into the midbrain, and terminate in the temporal lobe (Cope et al., 2015). Thus, hearing loss on one side results from dysfunction of crossed (contralateral) lateral lemniscus pathways. Contralateral hearing loss caused by ischaemic stroke due to pons infarction has been previously reported in patients with superior cerebellar artery syndrome (Murakami et al., 2005). Such pontine infarction typically causes ipsilateral cerebellar ataxia and contralateral appendicular sensory disturbance, as well as nystagmus, vertigo, and vomiting (Murakami et al., 2005).

Central ocular motor features [refixation saccades with normal VOR gain on the vHIT (Chen et al., 2014; Koohi et al., 2022; Curthoys et al., 2023)], skew deviation, and head tilt were clear indicators of brainstem involvement. Of note, eye recording technology aided in the assessment of subtle eye movement abnormalities in this patient (subtle left gaze-evoked nystagmus and the presence of significant “refixation” saccades despite normal VOR gain). The left abducens nucleus innervates the lateral rectus muscle. Due to the suspected damage to this nucleus (Figure 5), the patient's eye exhibited a drift back to the primary position when attempting to look left (gaze-evoked nystagmus), indicating an inability to maintain left lateral gaze.

The brain MRI identified an acute infarct involving the lateral lemniscus where the majority of the ascending auditory fibers cross over to the contralateral olivary complex or lateral lemniscus (accounting for the right-sided hearing loss).

In summary, our case serves to highlight that a distinction between central and peripheral causes of acute audiovestibular symptoms should be anchored on a clinical assessment rather than exclusively on negative early imaging. Thus, in patients presenting with acute audiovestibular abnormalities, it is important to first identify central neurological features (facial or appendicular sensory loss, dysphasia, dysphonia, diplopia, gait ataxia, etc.) and, where absent, perform a detailed neuro-otological assessment, particularly in patients with vascular risk factors. Diagnosis may be confirmed on delayed brain MRI, although this can be negative in patients with very small areas of ischaemia (Saber Tehrani et al., 2014).

## Patient's perspective

The patient was comforted by the swift diagnosis and thorough investigations conducted by the acute neurology team, which included specialists from the acute vestibular team. Subsequent referral to the neurophysiotherapy team allowed him to improve his general strength and fatigue. He has noted a marked improvement in his symptoms but recognizes the emotional impact these health challenges have imposed.

## Data availability statement

The original contributions presented in the study are included in the article/supplementary material, further inquiries can be directed to the corresponding author.

## Ethics statement

Written informed consent was obtained from the individual(s) for the publication of any potentially identifiable images or data included in this article.

## Author contributions

NeK: Conceptualization, Data curation, Formal analysis, Investigation, Resources, Validation, Visualization, Writing – original draft, Writing – review & editing, Project administration, Supervision. SH: Data curation, Formal analysis, Investigation, Validation, Visualization, Writing – review & editing. NaK: Data curation, Formal analysis, Investigation, Validation, Visualization, Writing – review & editing. DW: Data curation, Formal analysis, Investigation, Validation, Visualization, Writing – review & editing. D-EB: Data curation, Formal analysis, Investigation, Validation, Visualization, Writing – review & editing. DK: Conceptualization, Data curation, Formal analysis, Investigation, Project administration, Resources, Supervision, Validation, Visualization, Writing – original draft, Writing – review & editing.
